# MomMoodBooster Web-Based Intervention for Postpartum Depression: Feasibility Trial Results

**DOI:** 10.2196/jmir.2876

**Published:** 2013-11-01

**Authors:** Brian G Danaher, Jeannette Milgrom, John R Seeley, Scott Stuart, Charlene Schembri, Milagra S Tyler, Jennifer Ericksen, Whitney Lester, Alan W Gemmill, Derek B Kosty, Peter Lewinsohn

**Affiliations:** ^1^Oregon Research InstituteEugene, ORUnited States; ^2^Parent-Infant Research InstituteHeidelberg Repatriation HospitalHeidelberg Heights, VictoriaAustralia; ^3^Melbourne School of Psychological SciencesUniversity of MelbourneMelbourneAustralia; ^4^Depression and Clinical Research CenterUniversity of Iowa Hospitals and ClinicsIowa City, IAUnited States

**Keywords:** postpartum depression, Web-based intervention

## Abstract

**Background:**

Postpartum depression (PPD)—the most common complication of childbirth—is a significant and prevalent public health problem that severely disrupts family interactions and can result in serious lasting consequences to the health of women and the healthy development of infants. These consequences increase in severity when left untreated; most women with PPD do not obtain help due to a range of logistical and attitudinal barriers.

**Objective:**

This pilot study was designed to test the feasibility, acceptability, and potential efficacy of an innovative and interactive guided Web-based intervention for postpartum depression, MomMoodBooster (MMB).

**Methods:**

A sample of 53 women who satisfied eligibility criteria (<9 months postpartum, ≥18 years of age, home Internet access and use of personal email, Edinburgh Postnatal Depression Survey score of 12-20 or Patient Health Questionnaire score from 10-19) were invited to use the MMB program. Assessments occurred at screening/pretest, posttest (3 months following enrollment), and at 6 months follow-up.

**Results:**

All six sessions of the program were completed by 87% (46/53) of participants. Participants were engaged with the program: visit days (mean 15.2, SD 8.7), number of visits (mean 20.1, SD 12.2), total duration of visits in hours (mean 5.1, SD 1.3), and number of sessions viewed out of six (mean 5.6, SD 1.3) all support high usage. Posttest data were collected from 89% of participants (47/53) and 6-month follow-up data were collected from 87% of participants (46/53). At pretest, 55% (29/53) of participants met PHQ-9 criteria for minor or major depression. At posttest, 90% (26/29) no longer met criteria.

**Conclusions:**

These findings support the expanded use and additional testing of the MMB program, including its implementation in a range of clinical and public health settings.

**Trial Registration:**

Clinicaltrials.gov NCT00942721; http://clinicaltrials.gov/ct2/show/NCT00942721 (Archived by WebCite at http://www.webcitation.org/6KjYDvYkQ).

## Introduction

Postpartum depression has been defined to include any major or subsyndromal depression present at any time during the first year after delivery [1], and it is the most common complication of childbirth [2]. In terms of prevalence (10-20% of women) and severity, PPD lies between “baby blues” (less severe and quite common affecting 80% of women) and postpartum psychosis (more severe and less common affecting 0.1-0.2% of women) [1]. Left untreated, PPD has serious consequences [3]: for the mother, diminished well-being, feelings of failure, difficulties interacting with her infant, her family (partner’s mental health, relationship problems); and for her infant, compromised cognitive and psychosocial development [4-8] and increased risk of mental health difficulties even in adolescence [9].

PPD is related to a range of biopsychosocial and cultural factors [10]. Previous episodes especially during pregnancy [11] or family history of mental health problems, low social and emotional support, drug and alcohol abuse, past or present abuse [12], and major life stressors are all major risk factors for PPD [13]. Less salient risk factors include marital relationship difficulties, low income, unemployment, and obstetric factors and complications [14]. Issues encountered during the reproductive year also increase risk for PPD, premature birth, and subsequent hospitalization of the infant (including care in a neonatal intensive care unit) [15-17]. Culturally and linguistically diverse women, especially refugees, asylum seekers, and immigrants have increased risk [18].

There are also significant economic and social costs (eg, loss of productivity for mother and father, health care costs, personal and broader social and economic costs) to the community. For example, Dagher et al [19] reported that PPD was related to increased health care services use, which translated into higher costs to providers. Research from the United Kingdom indicates that costs associated with PPD are higher in high-risk women [20]. An Australian analysis estimated the cost of PPD and anxiety in mothers delivering in 2012 to be $500 million by the time the children reach 2 years of age [21].

Given the limited empirical evidence supporting use of antidepressant medication with PPD [1,22], there is a significant need to develop effective psychosocial treatment approaches. Recent meta-analyses of psychosocial interventions for PPD concluded that they have a moderate beneficial effect [23,24]. Treatment modalities have included counseling [25], interpersonal psychotherapy (IPT) [26,27], and cognitive behavior therapy (CBT). A wealth of research supports the effectiveness of CBT interventions for depression in general [28,29], and perinatal depression specifically. About two-thirds of depressed individuals receiving CBT remit with treatment [30] and also have a reduced risk of relapse [29]. These benefits appear to accrue particularly in individuals with mild to moderate depression [31]. Milgrom and her colleagues created and conducted a series of successful trials [32,33] using a face-to-face individual PPD treatment program based on an adaptation of the Coping with Depression course by Lewinsohn [33,34] that also included elements of IPT (provided content on interpersonal relationships, an opportunity for partners to become involved and provide input, and a focus on infants).

Although recent trials [35,36] have demonstrated that, within a collaborative care model for depression, women suffering from PPD can be screened within a stepped-care treatment protocol during visits to their health care provider [36,37], the data indicate that, overall, fewer than 50% of postpartum women receive help for their depression [38-40]. Many of the available PPD treatment approaches are office-based, which reduces their practicality for new mothers. In addition, patient-level barriers to the uptake of treatment include travel requirements, childcare, stigma, feelings of failure, poor understanding of depression or what help is available, and safety concerns about using prescription medications [41-44]. Provider-level barriers that discourage physicians and medical/clinic staff from becoming more fully involved in PPD screening and treatment include their lack of knowledge and skills due to insufficient training regarding depression and mental health, their fear of liability, the dearth of mental health treatment resources and flexible referral systems, and inadequate reimbursement [45-50].

Web-based PPD treatment may reduce both patient- and provider-level barriers to treatment uptake and thus extend the reach of helpful treatments to underserved mothers suffering from depression. For example, Web-based treatments can reduce feelings of stigma because participation is relatively anonymous and can be completed in women’s homes (thus avoiding travel) at times of their choosing without requiring childcare arrangements. Providers can recommend that women use an evidence-based Web-based PPD treatment program thus alleviating their concerns about training deficits and/or time required to provide treatment.

An increasing number of Web-based depression interventions have emerged [31,51-61]. The efficacy of these interventions has been demonstrated relative to control conditions in populations with elevated symptoms and, increasingly, in clinically diagnosed groups [62-66]. Face-to-face CBT has also been compared with Web-based CBT treatment. For example, Spek et al [60] found that both in-person and Web-based CBT interventions were superior to a waitlist control, that no significant differences were found between intervention modality, and that reductions in depressive symptoms were maintained at least 1 year after initiation of Web-delivered CBT. Similar results have emerged in other published comparisons [67,68]. Reviews of the available evidence [57,58,64,69] indicate that purely self-guided Web-based interventions benefit depressed individuals, but that effect sizes were enhanced when online programs were facilitated by a live coach [70]. Trained coaches have been shown to enhance the therapeutic alliance of Internet programs by providing low-intensity support [71] and increasing adherence to online mental health treatments [70,72].

Based on our review to date, there has been only one published report of the results of a Web-based depression intervention for postpartum women. In the randomized controlled trial (RCT) by O’Mahen et al based in England, 910 women with PPD symptoms (>12 on the Edinburgh Postnatal Depression Survey or EPDS [73]) were randomly assigned to either (1) a full-featured 11-sesssion Web-based behavioral activation intervention (N=462) contextualized for PPD (NetMums) that also included access to features of the popular NetMums website/online community, special chat access to parent-supporters and specialist health visitors, or (2) a treatment as usual condition (N=448) [74,75]. Although there was notable attrition at the 15-week follow-up (61% attrition in the intervention and 64% in the control), results showed significant benefits to the intervention versus the control condition. Among completers at follow-up, there was clinically significant improvement among 61% of women in intervention versus 41% in the control.

The present study reports on the Web-based MomMoodBooster (MMB) program based on Milgrom’s adaptation of the Coping With Depression Course (CWDC) [76] for postpartum depression [32,33,77] as well as an adaptation of the CWDC for Web-based delivery [78]. MMB was developed and pilot-tested by a multinational team from Oregon Research Institute (ORI), Parent-Infant Research Institute (PIRI) in Melbourne, Australia, and the Iowa Depression and Clinical Research Center (IDCRC). The Australian version of the program was localized for spelling (eg, it was rebranded to MumMoodBooster*)*, word choices, and selected videos. We described the formative research foundation for this MMB in a previous paper [79]. This report describes the outcome results of a feasibility trial of the MMB program.

## Methods

### Participants and Procedures

Participants (N=53) were recruited from two different research sites (n=27 from our US site in Iowa and n=26 from our Australia site in greater Melbourne). Prospective participants were identified via birth records, nurse/health professional referrals, online advertisements, and news stories to local university and hospital settings.

Upon receipt of a referral or direct contact from a prospective participant, each woman was contacted by a member of the research team to explain the study and obtain informed consent for participation. During the initial contact, a preliminary check of eligibility criteria was conducted. Preliminary screening criteria included <9 months postpartum, ≥18 years of age, home Internet access and use of personal email, and an EPDS score [73] from 12-20 or a Personal Health Questionnaire (PHQ-9) score [80] from 10-19. These ranges were chosen to identify women with mild to moderately severe depression. Women satisfying initial eligibility criteria were then mailed a Participant Information and Consent Form for their signature.

Women meeting initial screening criteria then completed a phone-administered Structured Clinical Interview for DSM-IV Disorders (SCID) [81,82] and the Hamilton Rating Scale for Depression (HRSD) [83-86] to evaluate the following exclusion criteria: current diagnosis of substance abuse, bipolar disorder or psychotic depression, and/or current treatment for depressive symptoms including antidepressant medication or psychotherapy. A participant’s endorsement of suicidal statements on assessments or to project staff triggered a suicide risk management protocol designed to determine the presence of current plans for self-harm, resulting in an offer of assistance and exclusion from participation in the study. Women who satisfied all inclusion and exclusion criteria were invited to participate in the study and were asked to complete the pretest assessment by visiting the secure research website. Women who did not meet eligibility criteria were offered treatment through the Infant Clinic (Australian site) and/or referral to other services as appropriate (US site). The research protocol and related informed consent procedures were reviewed and approved by the Human Research Ethics Committee of Austin Health in Australia and the Institutional Review Boards of both ORI and the University of Iowa.

Following enrollment, participants worked through the MMB program and received weekly phone calls from a personal coach (psychologist or graduate research assistant at the Australian site or a research assistant at the US site) who encouraged participants to use the program, to practice the recommended strategies, and to report their mood levels on a PHQ-9 assessment. Every effort was made to use the same personal coach for each participant on each call. The program automatically sent email reminders to encourage participants to log into the program.

### Measures

#### Overview

**Figure 1:**
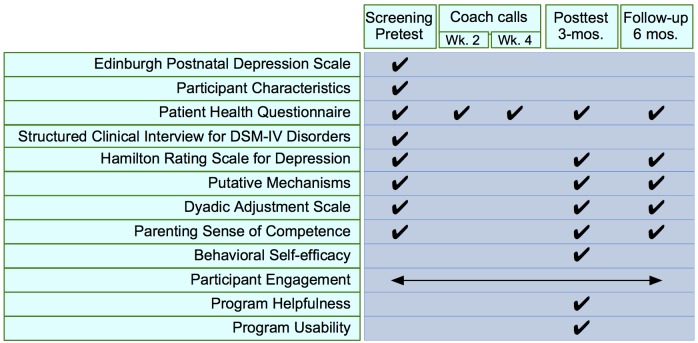
Measures by assessment point.

As described in Figure 1, assessments occurred at screening/pretest (corresponding to enrollment), a posttest (3 months following pretest), and follow-up (6 months following pretest). At posttest and follow-up, participants were asked to complete questionnaires both by visiting the secure website and completing another assessment by phone. By using the same phone assessor, we hoped to obtain a more sensitive measure of change. Expert phone assessors from our US research site provided assessors in our Australia site with systematic training (videoconferencing and reliability training using audio test cases) in the use of the SCID and HRSD. All phone-based assessments were recorded and reviewed.

#### Structured Clinical Interview for DSM-IV Disorders

Trained diagnostic interviewers conducted phone-based SCID interviews [81,82]. In order to minimize respondent burden, we used SCID Modules A-F, but we did not include the Somatoform, Eating Disorders, and Adjustment Disorder Modules.

#### Hamilton Rating Scale for Depression

Interviewers also administered the HRSD [27,83-86] by phone. Scoring is based on the sum of 24 items. The maximum overall score for the HRSD-24 is 69. For the current study, Cronbach alpha=.76.

#### Edinburgh Postnatal Depression Scale

During screening, participants were asked to complete the EPDS, a brief, simple self-rated, 10-item measure developed to screen for symptoms of postpartum depression [38,87]. Responses are rated from 0 to 3 and summed to yield the score with a maximum overall score of 30.

#### Participant Characteristics

We measured maternal age, delivery date and gestation, parity, education, history of previous treatment for depression, and household income.

#### Patient Health Questionnaire

Participants were asked to complete six separate PHQ-9 assessments from pretest, posttest, and follow-up. Personal coaches administered the PHQ-9 during phone calls that corresponded to Sessions 3 and 5 of the MMB program. These serial PHQ-9 assessments were used for program evaluation, to provide participants with a useful assessment of their status, and as an important safety check of participant status [80,88-90]. PHQ-9 scores showing a 5-point or greater escalation from pretest triggered a safety protocol, as did endorsement of the PHQ-9 suicidality item. The maximum overall score for the PHQ-9 is 27. For the current study, Cronbach alpha=.76. To evaluate the clinical significance of the intervention effects, we calculated the minimal clinically important difference (MCID [91]) based on Lowe et al [89], which represents a reduction in the PHQ-9 score of 5 points or greater. Thus, pretest-posttest changes on the PHQ-9 of ≥5 points represented a clinically important difference. For the current study, Cronbach alpha=.76.

#### Automatic Thoughts Questionnaire

Participants were asked to indicate how frequently over the previous week they had negative thoughts using the 30-item Automatic Thoughts Questionnaire (ATQ) [92,93] (eg, “My life is a mess”). Value options range from 0 to 4 (0=Not at all to 4=All of the time) with a maximum score of 120. For the current study, Cronbach alpha=.92.

#### Behavioral Activation for Depression Scale

We used the 25-item Behavioral Activation for Depression Scale (BADS) to measure changes in activation, avoidance/rumination, work/school impairment, and social impairment (eg, “I stayed in bed for too long even though I had things to do”) [94]. Value options range from 0 to 6 with a maximum score of 150. For the current study, Cronbach alpha=.83.

#### Dyadic Adjustment Scale

We assessed women’s relationships with their partners using the Dyadic Adjustment Scale-7 (DAS-7) [95], an abbreviated version of the Dyadic Adjustment Scale [96]. The general satisfaction score was calculated as the sum of all scores (maximum score=36). For the current study, Cronbach alpha=.85

#### Parenting Sense of Competence

We included the Parenting Sense of Competence (PSOC) efficacy scale [97] that asks the participant to describe her extent of agreement with 7 items designed to assess whether she is knowledgeable and competent in being a mother [98] (eg, “I honestly believe I have all the skills necessary to be a good mother to my baby”). Value options ranged from 1 to 6 (1=Strongly Disagree to 6=Strongly Agree) with a maximum score of 42. For the current study, Cronbach alpha=.90.

#### Behavioral Self-Efficacy

Based on the work of Bandura [99] and Maciejewski et al [100], we used 8 items to assess participant self-efficacy or confidence in being able to work with the program to reduce feelings of depression at pretest and posttest. The question asked was, “During the past week, including today, how confident are you in your ability to… (1) increase your daily pleasant activities?; (2) control your negative thinking?; (3) increase your positive thinking?; (4) get support when you need it?; (5) keep track of your mood?; (6) reduce tension using relaxation?; (7) set realistic goals for yourself?; and (8) manage your mood?” Value options ranged from 1 to 5 (1=Not At All Confident to 5=Very Confident). Self-efficacy score was computed as the mean across 8 items. For the current study, Cronbach alpha=.88.

#### Website Metrics

We used industry-standard website analytic tools and planned database flags recommended by Peterson [101] to track visit patterns including the date/time for each webpage viewed, which enabled us to unobtrusively measure visit frequency and duration. We also considered ways that participants were able to initiate interactions with the program (see Table 1) that shared similar characteristics, as in initiate interaction only (eg, play a video or tutorial), enter personal data into an activity (eg, typed in reasons into a list, completed a drag and drop activity, completed online activities as part of recommended homework), and personalized features of the program (eg, set goals for daily pleasant activities, updated tracking of mood and activities, uploaded personal pictures).

#### Personal Coach Call Metrics

Personal coaches also tracked the number and duration of calls with participants. After each call, personal coaches provided an impression of their working alliance with the participant (response options: 1=minimal, 2=partial, 3=good, 4=excellent) and the level of distraction during the call (response option: 1=none/limited, 2=some, 3=a lot). 

#### Program Helpfulness (Self-Report by Phone at Posttest)

We used open-ended items to ask participants to identify aspects of the program that were most helpful and least helpful. We also asked participants if they would recommend the program to other depressed postpartum women.

#### Program Usability (Self-Report Online at Posttest)

We obtained a quantitative measure of usability by asking participants to complete our adapted version of the System Usability Scale (SUS) [102,103], a 10-item scale that asked the participant to rate the degree to which she agreed (1=Strongly Disagree to 5=Strongly Agree) with positive and negative descriptions of a Web-based program (eg, “I think that I would like to use this website frequently”) [79]. The maximum score (indicating maximum usability) is 100. For the current study, Cronbach alpha=.80.

**Table 1:** Participant engagement activities in MomMoodBooster.

Activity	Function	Examples
List activities	Encouraged creation of personal lists to gain insight into their situation.	Lists of my pleasant activities, list of supporters, my reasons for wanting to feel better, my contributing factors, my high-tension situations, my warning signs.
Expand-collapse activities	Enabled exploration of additional detail on topics of interest.	FAQs, Myths & Facts, etc.
Drag & drop activity (see Figure 3)	Provided an interactive experience to more clearly distinguish between topics.	Activity focusing on the difference between extreme thoughts and everyday concerns.
Goal setting activity (see Figure 4)	Interactive series of steps to encourage selection of goals.	Activity designed to help the participant to choose (1) the number of pleasant activities to accomplish each day, and (2) which strategies to work on once the program had concluded.
Practice change activities	Homework tasks that were to be accomplished by each participant in their normal routine, the results of which could be shared with the personal coach.	Noticing and identifying a downward spiral, what started it and what happened; practice relaxation, making the most from pleasant activities by anticipating and savoring activities.
Online behavior tracking	Online tools used to capture participant data over time designed to encourage self-monitoring, to illuminate patterns, and to show progress.	Daily tracking of mood ratings and pleasant activities accomplished. These tracked data were also charted online.
Testimonial videos	Streaming videos of coping models who overcome barriers in order to make changes recommended in the program.	Other women’s experiences; asking for help, not worrying, doing more fun activities, mood patterns, or managing stress.
Animated tutorials (see Figure 2)	Animations used to provide an explanation for underlying models for change.	Tutorials showed downward mood spirals and how they can be interrupted at critical choice points.
Personalizing pictures	Enabled participants to personalize the appearance of the program, to make it feel like “their own” website.	Women could add 10 pictures of their choice to personalize the webpages of the MMB program.

**Figure 2:**
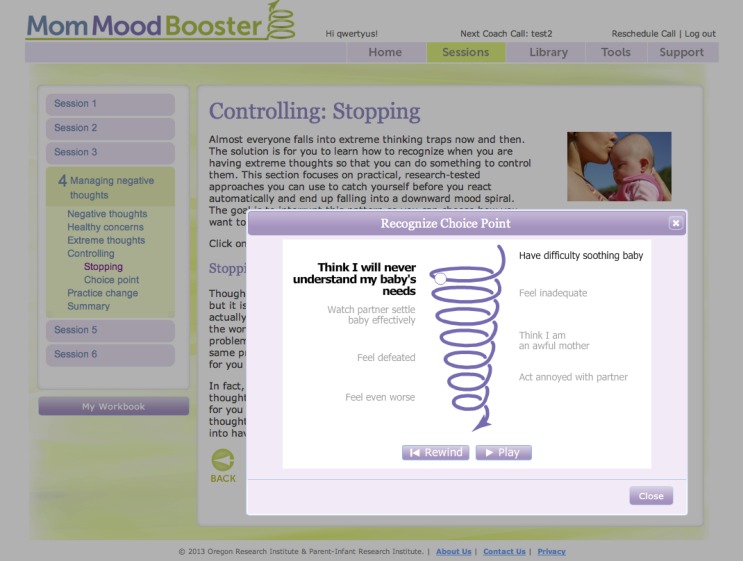
Animated tutorial engagement activity.

**Figure 3:**
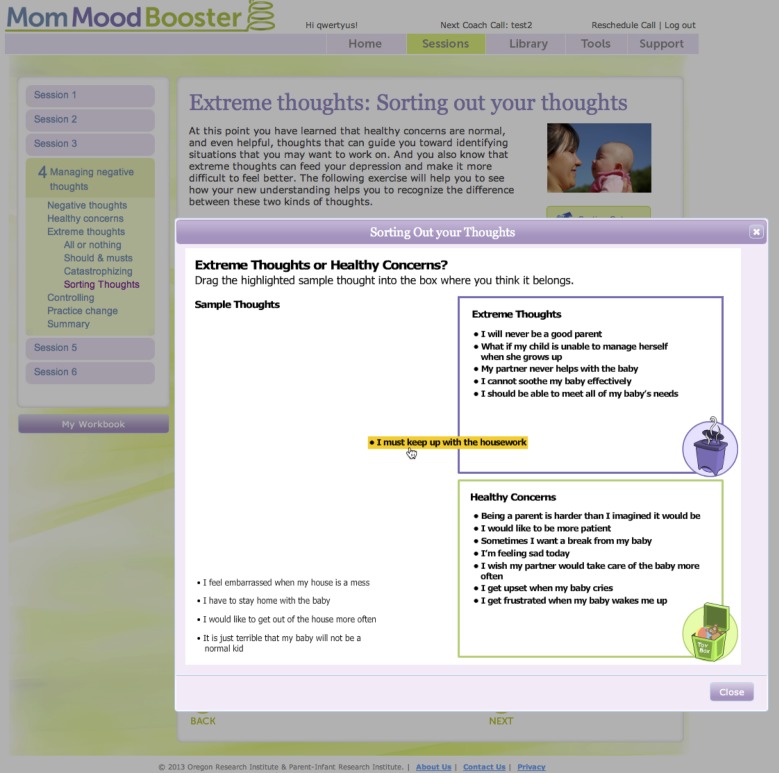
Drag & Drop engagement activity.

**Figure 4:**
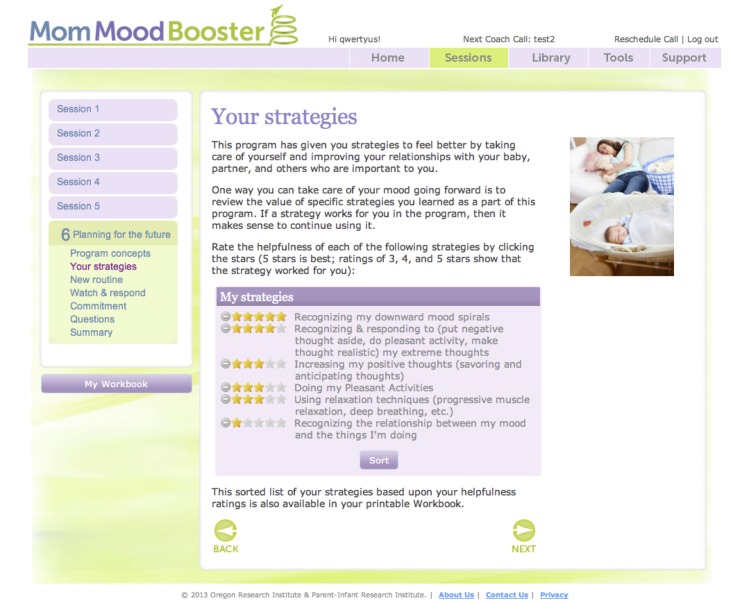
Goalsetting engagement activity.

### MMB Intervention

#### Web-Based Content

We developed the MMB program using an iterative formative research process that included focus groups and usability testing [79]. A detailed description of this process and a schematic depiction of the MMB program is available in our earlier publication [79]. The MMB program also includes three complementary websites: (1) a personal coach portal to enable coaches to review the progress each participant has made going through the program, (2) a simplified Partner Support website designed to provide participant partners with information about PPD and an overview of MMB, and (3) an administrative website that enabled research staff to monitor completion of the assessments and other elements of the research project. The MMB program was designed to be fully scalable and to run on PC and Mac computers using various current browsers without plugins or applications. Java scripting, HTML+CSS, and Dynamic HTML were used to deliver interactive content.

The program consisted of the following six sequential sessions with each successive session becoming available weekly. Sessions were as follows: (1) Getting Started, (2) Managing Mood, (3) Increasing Pleasant Activities, (4) Managing Negative Thoughts, (5) Increasing Positive Thoughts, and (6) Planning for the Future. Each session opened with an autoplay host video that introduces the session goals. Webpages delivered content using text, programmed interactions, animations, and videos to present program content. Tunnel architecture [104] was used to guide participants through the six sessions. While each successive program session could be accessed weekly, the schedule was flexible in that participants could take an additional week to complete any session. Coach calls corresponded to each program week. Thus, it was possible for the MMB program to be completed in 6-12 weeks with 6-12 coach calls.

The program includes a number of features designed to encourage participant engagement and behavior change (see Table 1 and Figures 2-4). For example, each day the program encouraged participants to enter ratings of their mood and to note the number of pleasant activities they engaged in. They were also able to type in personal lists, view videos and animations, and access a library of relevant articles on communication skills, getting support, managing stress, managing time, solving problems, sleep and caring for baby, baby’s needs, and relationship with partner.

Because social isolation and stigma are common in this population, MMB includes a private peer-based Web forum in which mothers can post messages as well as read and interact with the messages of other program participants. Finally, the program could be used by participants to send an email invitation to their partner encouraging them to visit a separate MMB informational website that described postpartum depression, the MMB program, and the important role of partners play [105,106].

As research shows, receiving email reminders can help to encourage greater adherence with Web-based interventions [31], MMB participants were sent automated email reminders to encourage their engagement with the Web-based program as well as to prompt them to complete the online assessments. Participants were able to access the online program for 6+ months following enrollment.

#### Personal Coach Calls

The entire program was facilitated by a series of phone calls with a personal coach. All coaches were graduate research assistants or research psychologists who had received training in the content of the MMB program, their roles as coaches versus therapists/counselors, and in their data collection responsibilities. Coach training started with a guided tour of the MMB program and its coach portal that summarized participant use of the features of each session. This was followed by a videoconference that included review of the coach manual that contained detailed scripts for each call, the coach data collection responsibilities, and a discussion of the role of the coach and the rationale for making calls (ie, to provide a human voice behind the automated program, to help each participant problem solve possible barriers to using the program, and to encourage program use). All coach calls were audiorecorded, and a subset was selected to monitor fidelity of implementation as well as reliability of phone-administered assessments.

### Statistical Analysis

Changes in PHQ-9 scores across time were evaluated using an unconditional growth model nesting repeated measures within individuals. This multilevel model includes time as the only predictor, coded as the number of weeks since the pretest assessment, and allows the number and spacing of measurement occasions to vary across persons [107]. A self-efficacy score was computed as the mean across 8 items. Pretest to 3-month posttest and pretest to 6-month follow-up comparisons on the ATQ, BADS, DAS-7, PSOC, and self-efficacy were evaluated using paired samples *t* tests.

All analyses involved an intent-to-treat approach whereby missing data were addressed in one of two ways recommended by Schafer and Graham [108]. We used a model-based maximum likelihood procedure in the analysis of PHQ-9 data in which parameter estimates were computed based on all available raw data. We used detailed data on participant engagement as person-level predictors of the linear and quadratic slope parameters specified in the unconditional PHQ-9 growth model. We also used the multiple imputation procedure in SPSS version 21 to account for missing data for our analysis of the ATQ, BADS, DAS-7, PSOC, and self-efficacy outcomes. Our multiple imputation procedure was fully conditional and used the iterative Markov chain Monte Carlo method to generate 20 complete datasets using all outcomes across time as predictors of missing values. The imputation model for each variable was a linear regression and included a constant term and main effects of predictor variables. Paired samples *t* tests were conducted for each measure across each of the 20 imputed datasets and reported pooled estimates in the results. To supplement tests of statistical significance, we computed partial point-biserial *r* as a measure of effect size in accordance with Rosenthal [109]. Partial point-biserial *r* was defined as √(*t* ^2^ / (*t* ^2^ + df)); small effect size=0.14, medium effect size=0.36, and large effect size=0.51 [110].

## Results

### Participant Characteristics and Study Attrition

Of the women who started the study, two were withdrawn because of concerns regarding self-harm and one woman withdrew of her own volition because she reported that she was feeling better and no longer wanted to be in the study. The resulting sample of 53 study participants had a mean age of 31.9 years (SD 5.1), a mean of 39.1 weeks (SD 2.4) gestation when their baby was born, and a mean number of 2.0 (SD 1.1) children. Mean baby age at the pretest was 5.5 months (SD 2.9). Participants were relatively well educated (14/53, 26% reported having graduate or postgraduate degrees) and 59% (31/53) reported annual family income of at least $60,000. Based on the SCID at screening, 49% (26/53) met criteria for DSM-IV major depressive disorder. Pretest characteristics of participants are presented in Table 2.

Of the remaining participants, 87% (46/53) completed all six sessions in the program. Posttest data were collected from 89% (47/53) on all key measures with the exception of the HRSD (45/53, 85%). Follow-up data at 6 months were collected from 87% of women (46/53). The extent to which attrition threatened the external validity of the study was evaluated using contingency table analyses and *t* tests. Overall attrition was 13% (7/53) from pretest to 6-month follow-up. Attrition was not associated with demographic characteristics or pretest values on the outcome measures. Given the minimal rates of missing data and the low likelihood of bias due to attrition, maximum likelihood estimation and multiple imputation procedures were appropriate for modeling potential intervention effects. Note that imputation was used to handle both types of missing data (ie, fully missing and “present” but with partial data).

**Table 2:** Selected participant characteristics at pretest (N=53).

Characteristics		n	%
**Baby’s gender**			
	Male	28	53
	Female	25	47
**Pregnancy was a multiple birth**			
	Yes	2	4
	No	50	94
	No answer	1	2
**Marital status**			
	Married	43	81
	Widowed	1	2
	Divorced	1	2
	Separated	2	4
	Single	6	11
**Education**			
	< High school	5	9
	High school	6	11
	GED/certificate level	5	9
	Associates degree/advanced diploma	2	4
	Bachelor degree	17	32
	Master/graduate degree	5	9
	Doctoral/postgraduate degree	9	17
	Other	3	6
	No answer	1	2
**Annual family income**			
	Up to $20,000	4	8
	$20,001-$40,000	9	17
	$40,001-$60,000	5	9
	$60,001-$80,000	14	26
	>$80,000	17	32
	No answer	4	8

### Primary Depression Outcomes

#### Patient Health Questionnaire Scores

As shown in Table 3, PHQ-9 scores decreased from pretest (mean 12.6, SD 4.1) to posttest (mean 5.0, SD 4.4) and the 6-month follow-up (mean 4.2, SD 3.9). Changes from pretest were statistically significant (*P*<.001) with large effects at posttest (partial *r*=.77) and 6-month follow-up (partial *r*=.82). In terms of clinical significance, at pretest, 55% (29/53) participants met PHQ-9 criteria for minor or major depression. At posttest, 90% (26/29) no longer met these PHQ-9 criteria. Results also indicated that 77% (36/47) of the participants experienced a minimal clinically important difference (ie, ≥5 point decrease) in their PHQ-9 depression scores from pretest to posttest.

**Figure 5:**
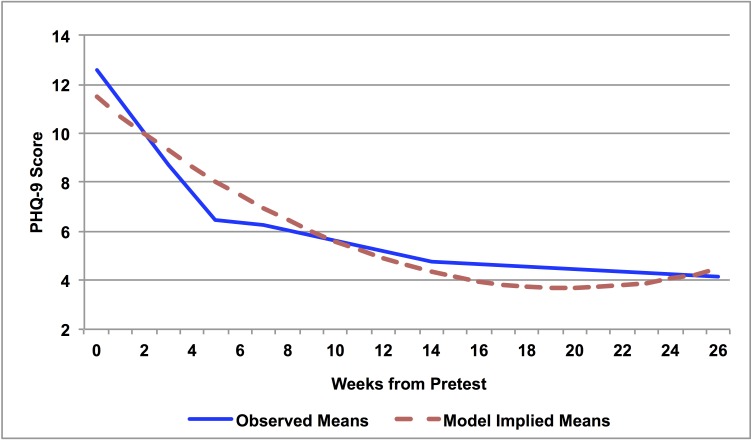
Longitudinal trajectories of the Patient Health Questionnaire-9 scores from pretest through 6-month follow-up.

Figure 5 depicts the observed and model-implied trajectory of PHQ-9 scores from pretest through the 6-month follow-up. A visual inspection of the data and a likelihood ratio (LR) test suggested that including a linear and quadratic growth parameter resulted in significantly better fit compared to a linear-only model (LR statistic with 2 degrees of freedom=36.79, *P*<.001). The statistical model that included linear and quadratic growth (-2 log-likelihood=1429.06, Akaike information criterion=1437.06, Bayesian information criterion=1451.25) implied an average pretest PHQ-9 score of 11.49 (SE 0.48), which decreased over time indicating a significant improvement in participant depression. Specifically, the model revealed a significant initial linear decrease (estimate=-0.79, SE 0.07, *P*<.001, partial *r=*.61) that significantly decelerated over time (estimate=0.02, SE 0.002, *P*<.001, partial *r*=.57). We also tested for differential trajectories in PHQ-9 scores between US and Australian participants by adding the main effect of region and the time by region interactions to the unconditional growth model described earlier. None of these parameters were statistically significant (*P*>.50), suggesting similar PHQ-9 trajectories between US and Australian participants.

#### HRSD Scores

As noted in Table 3, HRSD scores also decreased from pretest (mean 16.9, SD 6.9) to posttest (mean 7.0, SD 5.6) and the 6-month follow-up (mean 6.6, SD 6.8). Changes from pretest were statistically significant (*P*<.001) with large effects at posttest (partial *r*=.75) and 6-month follow-up (partial *r*=.71).

**Table 3:** Outcome results (mean is pooled mean; SD is average standard deviation across 20 imputed datasets).

Measure	Pretest	Posttest (3 mos.)	Pretest compared to posttest			Follow-up (6 mos.)	Pretest compared to follow-up		
	Mean (SD)	Mean (SD)	*t* (df=52)	*P*	Partial *r*	Mean (SD)	*t* (df=52)	*P*	Partial *r*
PHQ-9^a,c^	12.6 (4.1)	5.0 (4.4)	8.66	<.001	.77	4.2 (3.9)	10.43	<.001	.82
HRSD^a,d^	16.9 (6.9)	7.0 (5.6)	8.28	<.001	.75	6.6 (6.8)	7.28	<.001	.71
ATQ^a,e^	23.7 (12.0)	11.2 (10.7)	6.29	<.001	.66	10.8 (13.9)	4.95	<.001	.57
BADS^b,f^	78.4 (18.4)	103.9 (19.3)	-8.73	<.001	.77	105.6 (22.3)	-7.13	<.001	.70
PSOC^b,g^	2.9 (1.1)	3.6 (1.0)	-5.63	<.001	.62	4.0 (1.0)	-5.44	<.001	.60
Self-efficacy^b^	1.6 (0.7)	2.4 (0.8)	-4.32	<.001	.51	2.6 (1.0)	-6.61	<.001	.68
DAS^b,h^	22.0 (6.8)	22.5 (7.1)	-0.40	.689	.06	24.0 (8.8)	-1.78	.077	.24

^a^Lower score is better.

^b^Higher score is better.

^c^PHQ-9—Patient Health Questionnaire.

^d^HRSD—Hamilton Rating Scale for Depression.

^e^ATQ—Automatic Thoughts Questionnaire.

^f^BADS—Behavioral Activation for Depression Scale.

^g^PSOC—Parenting Sense of Competence Scale.

^h^DAS—Dyadic Adjustment Scale.

### Secondary Outcomes and Putative Mechanisms of Change

Table 3 also provides descriptive statistics and the pretest to posttest and pretest to 6-month follow-up comparisons on the ATQ, BADS, DAS-7, PSOC, and self-efficacy measures. Statistically significant and large effects from pretest to posttest were obtained for the ATQ (partial *r*=.66), BADS (partial *r*=.77), PSOC (partial *r*=.62), and self-efficacy (partial *r*=.51). From pretest to 6-month follow-up, statistically significant and large effects were obtained on the ATQ (partial *r*=.57), BADS (partial *r*=.70), PSOC (partial *r*=.60), and self-efficacy (partial *r*=.68). Measures of the DAS from pretest to posttest and follow-up did not show significant change.

### Website Engagement and Program Usability

Unobtrusive program use data indicated that mothers were engaged with the program: visit days (mean 15.2, SD 8.7); number of visits (mean 20.1, SD 12.2); total duration of visits in hours (mean 5.1, SD 1.3); and number of sessions viewed out of six (mean 5.6, SD 1.3). A total of 96% (51/53) of participants kept track of their daily mood ratings at least once (mean 38.1 days tracked, SD 25.6) and 92% (49/53) tracked the pleasant activities they wanted to accomplish each day (mean 29.2 days tracked, SD 23.7). The website forum provided the opportunity to post and view content; 38% (19/53) of mothers (mean 1.5, SD 2.9) posted forum content, and 74% (39/53) (mean 30.2, SD 26.8) viewed content. In addition, the MMB partner support website was accessed by 34% (18/53) of the participants’ partners. Results on the System Usability Scale [111,112] administered at posttest provide a quantitative measure of program ease of use. The mean System Usability Scale score was 84.4 (SD 11.6, range 52.5-100), which translates to a usability grade of “A” for the MMB program.

We explored associations between engagement and trajectories of PHQ-9 scores within a series of conditional growth models. These models included program engagement indicators as a composite measure as well as separately. Overall, the statistical models implied that program engagement was associated with additional decreases in PHQ-9 from pretest through follow-up. Specifically, the composite measure of program engagement was significantly related to the PHQ-9 linear slope parameter (estimate=-0.24, *P=*.021, partial *r=*.37). The linear slope parameter was also significantly related to the number of visit days to the program (estimate=-0.02, *P=*.043, partial *r=*.31) and selected engagement activities (see Table 1): the proportion of list activities completed (estimate=-0.02, *P=*.006, partial *r=*.43) and the proportion of activities engaged in that involved entering personal data (estimate=-0.01, *P=*.008, partial *r=*.44). Interestingly, the overall duration of program use was not significantly associated with the trajectories of the PHQ-9 scores.

### Personal Coach Calls

A total of 98% (52/53) of women agreed to receive personal coach calls. Coaches made a mean of 5.65 calls to each assigned participant (N=52; SD 1.58, Min=1; Max=9). Mean total contact duration per participant summed over all calls was 96.99 minutes (N=52; SD 49.21; Min=6.10; Max=212.07). Personal coaches reported, on average, that they had a good working alliance with participants (mean 3.07, SD 0.44) and reported low levels of distraction during the calls (mean 1.26, SD 0.35). 

### Participant Satisfaction

At posttest, participants reported being quite satisfied with MMB features (mean 3.3, SD 0.4 on a 4-point scale: Not at all satisfied to Very satisfied), and they rated personal coach calls as being helpful (mean 3.4, SD 0.9 on 4-point scale: Not at all helpful to Very helpful). Responses to open-ended questions about satisfaction are noted in Table 4.

### Use of Other Programs

At posttest we also asked participants “Since you enrolled in the MomMoodBooster program 3 months ago, which of the following products or programs have you used to manage your mood?”. A total of 30 out of 48 participants reported as follows: 12 (25.0%) read self-help books, 7 (14.6%) took medication for depression, 6 (12.5%) participated in an individual treatment program, 3 (6.3%) used hypnosis or acupuncture, 1 (2.1%) participated in a group treatment program, 1 (2.1%) participated in another Internet treatment program, and 30 (62.5%) participants indicated they had not participated in any other programs/products. Use of other programs for mood management was not significantly associated with the trajectories of the PHQ-9 scores.

**Table 4:** Participant comments on program satisfaction.

Question	Comments
Q1: In what ways did you find the Mum/MomMoodBooster program most helpful?	Support by phone, private time to do it
	Forced me to think about myself, focus on positive thinking helpful overall
	Valuable reassurance especially as can't get out
	Found it helpful in that feel more equipped to manage mood and emotions - online format is great as it allows easy access no matter what time of day
	Info was fine seemed very slow—sense of obligation was helpful—threat of phone coach calling forced to think about improving mood; to do list kind of person
	Phone calls to help keep you on track and tracking mood and activities so you can identify patterns
	Like how tasks were broken down into steps—strategies felt like they were achievable
	Gave permission to not have focus 100% be on the baby—to do something for self
Q2: In what ways did you find the personal coach calls to be helpful?	Really good at normalizing situations—also the flexibility of the coach (if baby cries, etc) was reassuring
	Reaffirmed things in the course, someone to talk to, to make sure you’re on track —not isolated—good that someone was going to call—something to look forward to—someone was going to ask you how you're doing with program—motivated to do program—sharing
	Help me remember to log in
	“Personal” feeling rather than website but content nothing new/warm
	Felt someone was caring
	Calls tie the whole program together act as a “check-in” for how feeling, review the materials from session
	Makes you accountable—keep going with session—would be easy to leave it for next week if no coach calls—helpful to talk through the information and clarify certain points

## Discussion

### Principal Findings

Pilot study results described in this report—when combined with results of our formative research [79]—provide comprehensive evidence supporting MomMoodBooster, an innovative Web-based intervention for postpartum depression. Pilot study participants, a clinical sample of 53 women recruited from the United States and Australia, were very engaged with the MomMoodBooster program: 87% completed the 6-month follow-up assessment, they viewed an average of 5.6 out of the 6 sessions, spent an average of more than 5 hours using the program, and spent an average of more than 95 minutes on personal coach calls. Their average number of 20.1 program visits compares quite favorably to results reported for many other Web-based depression interventions [113]. Participants also reported positive ratings regarding program usability, which was mirrored in their favorable ratings and comments regarding the program, including coach calls.

It is important to note that the relationship between participant engagement in the program and depression outcomes warrants further analysis as our measure of program use duration was not significantly associated with improvement in depression as measured by trajectories of the PHQ-9 scores. Elsewhere [114] we have recommended that there may not be simple dose:response relationships between engagement and outcome and that composite measures incorporating several dimensions of program usage need to be explored in this regard.

Over the course of the program, participants showed significant improvements on clinician-rated HRSD and self-reported PHQ-9 assessments, and they sustained those improvements over the 6-month follow-up period. Fully 77% reported experiencing clinically important improvement in their PHQ-9 scores. Putative mechanisms of change showed corresponding improvements.

Our 13% participant attrition is slightly higher than what has been reported for telephone-delivered therapies [113], and notably lower than the 25% to 50% reported in face-to-face psychotherapy and the sizable attrition rates reported in self-help Internet interventions [115]. Importantly, attrition was much lower than the nearly 60% attrition rate reported in a published paper on a Web-based intervention with women with PPD [74] and a third of that reported by Milgrom in her group-based CBT intervention for postpartum depression [33], the treatment approach embodied in the MMB program.

We believe that the highly encouraging results for participants using the MMB program were associated with three factors: (1) our adaptation and contextualization to PPD of Lewinsohn’s Coping with Depression Course, as embodied in Milgrom’s work, (2) MMB’s online engagement activities that encouraged participants to be actively involved, to spend time, and to follow treatment recommendations, and (3) personal coach calls that provided a key element of supportive accountability, which encouraged engagement and follow-through.

There are several study limitations that should also be noted. For example, we used a quasi-experimental design without a randomized controlled condition, thus we were not able to control for potential threats to the internal validity such as biases due to selection or maturation effects. In addition, our relatively small sample size may limit the generalizability of the study findings. We also recruited a convenience sample, which may not be representative of depressed postpartum women, generally. In addition, participants were relatively well educated and had a relatively high socioeconomic status. Finally, we did not assess the maintenance of the treatment benefits beyond 6 months.

### Next Steps

We agree with the conclusion expressed by Lewis et al [116]: “Given the time, cost, and childcare constraints of traditional interventions for postpartum depression, evaluations of new and innovative interventions are needed.” Based upon the promising results of our pilot study, we believe that the Web-based MomMoodBooster program represents just such an innovative treatment option. Next steps worthy of consideration include additional research. For example, controlled research is needed to evaluate MMB compared to alternative approaches when implemented within extant treatment programs based in real-world settings, such as in telephone-administered treatment programs [117-119], depression care management programs [120], nurse home visitations to pregnant and postpartum women [121], and in depression treatment provided in physician offices [35,36]. MMB would seem to be particularly appropriate within a stepped-care model as it could offer a low-cost, high-reach option as a preliminary treatment step [36,37] and/or in conjunction with other, more intensive “high-touch” treatments. Additional research might also examine the role of the personal coach. For example, rather than using research staff as coaches, it would be useful to test the use of endogenous providers as coaches. And since the cost and feasibility of providing 6 scheduled personal coaching calls may limit implementation opportunities, additional research might consider ways to provide fewer coach calls or provide a stepped-care approach that would tailor calls to the expressed interests of the recipient.

It would also be helpful to determine how program content might be adapted and delivered to reach low-income and minority postpartum depressed women by accommodating cultural differences [119], learning styles, and preferences in terms of tools/platforms to access program content (eg, use of smartphones is closing the “digital divide” [122,123]).

Finally, MMB could be expanded to include content on antenatal depression and/or content to enhance mother:infant interactions, two under-recognized and often untreated problems [45, 46] that have profound effects on maternal and infant well-being and health. In addition to being a risk factor for PPD, antenatal depression is related to more frequent pre-eclampsia [124], preterm birth [125], low birth-weight [126], and adverse obstetric outcomes [127]. It also diminishes capacity for maternal self-care as it can be accompanied by inadequate nutrition, drug and alcohol abuse, and poor prenatal clinic attendance, all of which can further compromise the health of mother and baby [128,129]. Because research shows that treating postpartum depression does not improve poor mother:infant interactions, which results in risk to maternal and infant well-being and health [1,130,131], then additional program content might be included in MMB in order to address this important area.

## Abbreviations

ATQ: Automatic Thoughts Questionnaire
BADS: Behavioral Activation for Depression Scale
CBT: cognitive behavioral therapy
CWDC: Coping With Depression Course
DAS-7: Dyadic Adjustment Scale (7-item version)
EPDS: Edinburgh Postnatal Depression Survey
HRSD: Hamilton Rating Scale for Depression
IDCRC: Iowa Depression and Clinical Research Center
IPT: interpersonal psychotherapy
MMB: MomMoodBooster (MumMoodBooster)
NHMRC: National Health and Medical Research Council (Australia)
ORI: Oregon Research Institute
PHQ-9: Patient Health Questionnaire (9-item version)
PIRI: Parent-Infant Research Institute (University of Melbourne)
PPD: postpartum depression
PSOC: Parenting Sense of Competence scale
SUS: System Usability Scale
SCID: Structured Clinical Interview for DSM-IV Disorders
